# Comparing impacts of the COVID-19 pandemic on training of public health Specialty Registrars starting before or after its onset

**DOI:** 10.1016/j.puhip.2022.100351

**Published:** 2022-12-17

**Authors:** S. Sisodia, S. McGill, M.S. Evans, G. Brough, U. Okereke, T.J. Dunn

**Affiliations:** a Division of Epidemiology and Public Health, Nottingham University, Nottingham, NG7 2QL, UK; b Centre for Environmental Health and Sustainability, University of Leicester, Leicester, LE1 7RH, UK; c Global Operations, UKHSA, Nobel House, 17 Smith Square, London, SW1P 3HX, UK; d Academic Unit of Population and Lifespan Sciences, School of Medicine, The University of Nottingham, Clinical Sciences Building, Nottingham City Hospital Campus, Hucknall Road, Nottingham, NG5 1PB, UK; e NHS England and NHS Improvement, Midlands, Seaton House, City Link, London Road, Nottingham, NG2 4LA, UK

**Keywords:** Public health, Specialty training, Covid-19, Mental health, Education

## Abstract

**Objectives:**

To capture and compare the differences in experiences of public health Specialty Registrars who commenced training prior to the COVID-19 pandemic (pre-pandemic Registrars) and those who commenced training during the pandemic (post-pandemic Registrars).

**Study design:**

This is a mixed methods study comprising a cross-sectional survey and participatory action research.

**Methods:**

A questionnaire of 10 open and 5 closed questions exploring participants experience of training during the pandemic was sent to East Midlands Specialty Registrars. Thematic analysis and double coding were undertaken, coded based on pre- or post-pandemic Registrar status. Participatory action research was then undertaken in 2 rounds with 2 groups, based on pre/post-pandemic status to consolidate themes.

**Results:**

The survey was completed by 17 Registrars (8 pre-pandemic, and 9 post-pandemic) and 19 Registrars took part in participatory action research. The findings showed pre-pandemic Registrars noted the importance of negative impacts on their mental health whilst post-pandemic Registrars were more positive and felt well supported in their training.

**Conclusions:**

There is a stark difference in the impact of the pandemic for Registrars who started training before compared to during the pandemic. The training programme was not resilient to the impact of the pandemic. Robustness could be increased by encouraging early leadership experience and providing wellbeing support, particularly for post pandemic Registrars now and in future.

## 1 Introduction

The COVID-19 pandemic has impacted the training of public health Specialty Registrars [1]. Public health training lasts a minimum of 48 months in the UK, and individuals from medical and non-medical backgrounds can apply. It has a reputation for developing highly skilled scientists, practitioners, and decision-makers [2]. The early phase of the pandemic dramatically impacted training in terms of content, workload, pace, level of supervision, and online working [1]. As the pandemic continues, new public health Registrars join training and may experience different challenges than those who started before the pandemic.

Concerns have been raised through a General Medical Council (GMC) report [3], highlighting the challenges faced by those training in medical specialities during the pandemic. There is a need to understand how training in Public Health Medicine has changed during the pandemic, and indeed whether the current training environment is able to produce public health professionals with the breadth of experience required to respond to the challenges of a post-pandemic world [2,4].

We seek to address two key aims:

1. To understand the experiences of public health Registrars based in the East Midlands engaged in the protracted COVID-19 pandemic response; and
2. To compare the perceptions and experiences of Registrars who commenced their training before the pandemic (pre-pandemic Registrars) with those who started after the pandemic (post-pandemic Registrars).

At the start of the pandemic, colleagues conducted a modified Delphi group to understand how the start of the pandemic challenged public health training in the East Midlands region of England. This found that Registrars had a firm identity as public health professionals, variable experiences of training, and had to adapt to rapid changes in work-life balances [1]. Our research aims to build on previous work by comparing experiences of pre- and post-pandemic Registrars.

## 2 Methods

### 2.1 Sample

In December 2021 there were 40 Public Health Registrars enrolled within the East Midlands deanery. Approximately 50% are medically qualified, with the remainder from allied health professions and public health practice.

### 2.2 Survey

The online survey was distributed to the Registrar cohort in December 2021–January 2022 (Appendix 1).

This consisted of ten open questions relating to training experiences, followed by a section on demographic variables that was developed from the preceding research [1]. An additional question about mental health was included based on findings from a recent GMC survey [3].

Responses were collected on Microsoft Forms and divided into those provided by pre- and post-pandemic Registrars, allowing for a comparative analysis between these two groups [5,6]. Personal identifiers were removed from the dataset by SM. All transcripts were analysed thematically [7] and double coded by SS, SM and TD. A master code book was created, and codes grouped into protothemes which were then presented and discussed at participatory action research groups.

### 2.3 Participatory action research groups

**Fig. 1:**
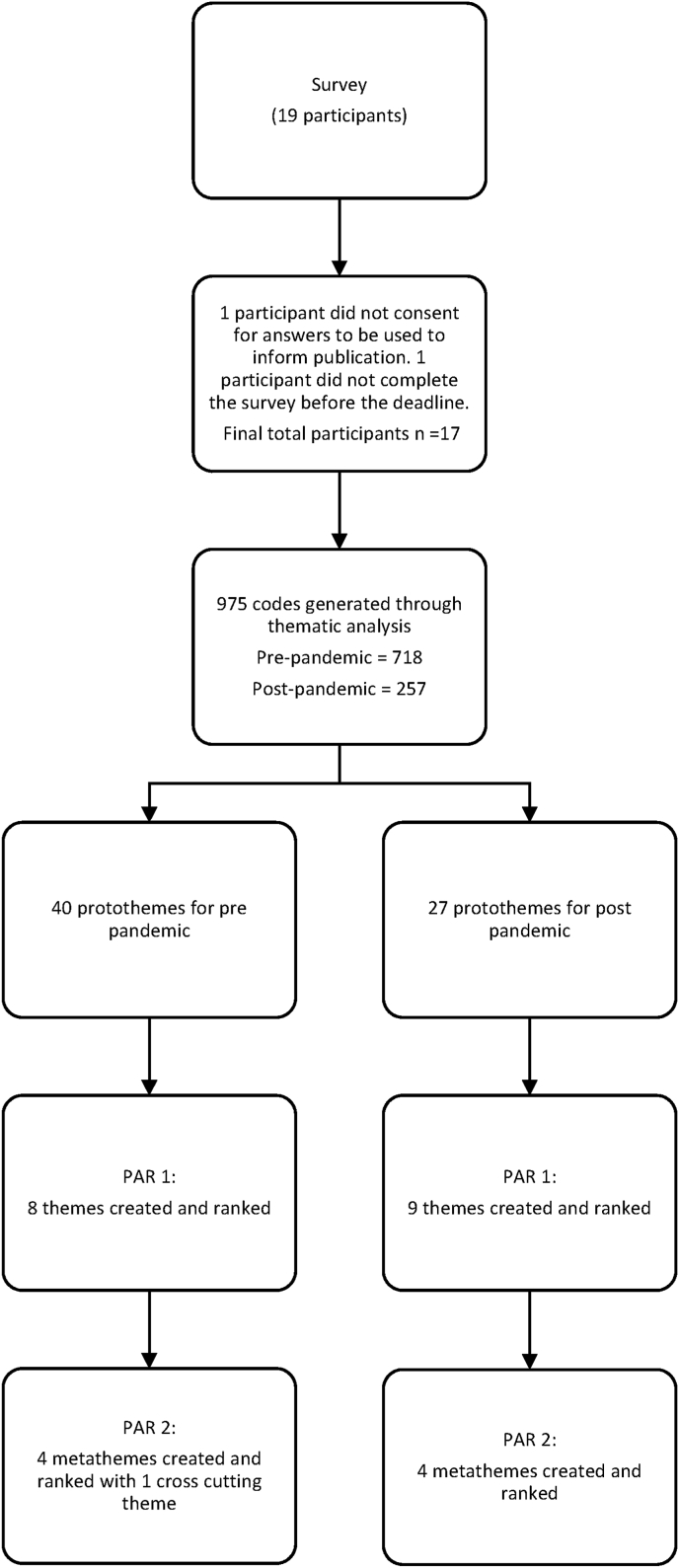
Theme generation.

Two concurrent participatory action research (PAR) groups (one consisting of Registrars who started training before the onset of the pandemic, and one with Registrars who started after) were conducted using Microsoft Teams on February 2, 2022. In round 1 of PAR, protothemes were condensed to create a ranked list of up to 10 themes. In round 2, themes were further amalgamated to a shorter list. This process is summarised in Fig. 1. The two groups were conducted independently, with authors participating. The final themes were shared with the Registrar cohort to increase validity, with no objections raised.

Participation in each round was voluntary, with participants providing written and verbal consent. Data was held securely in line with the Data Protection Act [8].

## 3 Results

### 3.1 Characteristics of participants

The survey was completed by 19 Registrars. One participant did not consent to their response being used in the study and one did not complete the questionnaire within the deadline. The final survey analysis consisted of 17 responses (see Table 1), with eight pre-pandemic and nine post-pandemic Registrars. Ethnicity was reported as “White” or “Non-White” to preserve anonymity of minority ethnic participants, and sex was not collected to further ensure that male ethnic minority participants (a minority in the cohort) could not be identified. PAR rounds 1 and 2 were attended by 19 participants (nine pre-pandemic and 10 post-pandemic).

**Table 1:** Characteristics of participants.

Characteristic	Number (%)
**Phase of training**	
1	10 (59%)
2	7 (41%)
**Background**	
Medical	10 (59%)
Background other than medicine	7 (41%)
**When joined training programme**	
Pre pandemic	8 (47%)
Post pandemic	9 (53%)
**Ethnicity**	
White	15 (88%)
Non-White	2 (12%)
**Age**	
<35	10 (59%)
>35	7 (41%)

### 3.2 Survey findings (protothemes)

Initial thematic analysis revealed 718 codes for the pre-pandemic group (amalgamated into 40 protothemes) and 257 for the post-pandemic group (amalgamated into 27 protothemes) (Appendix 2).

### 3.3 PAR 1 findings (themes)

#### 3.3.1 Pre-pandemic

**Fig. 2:**
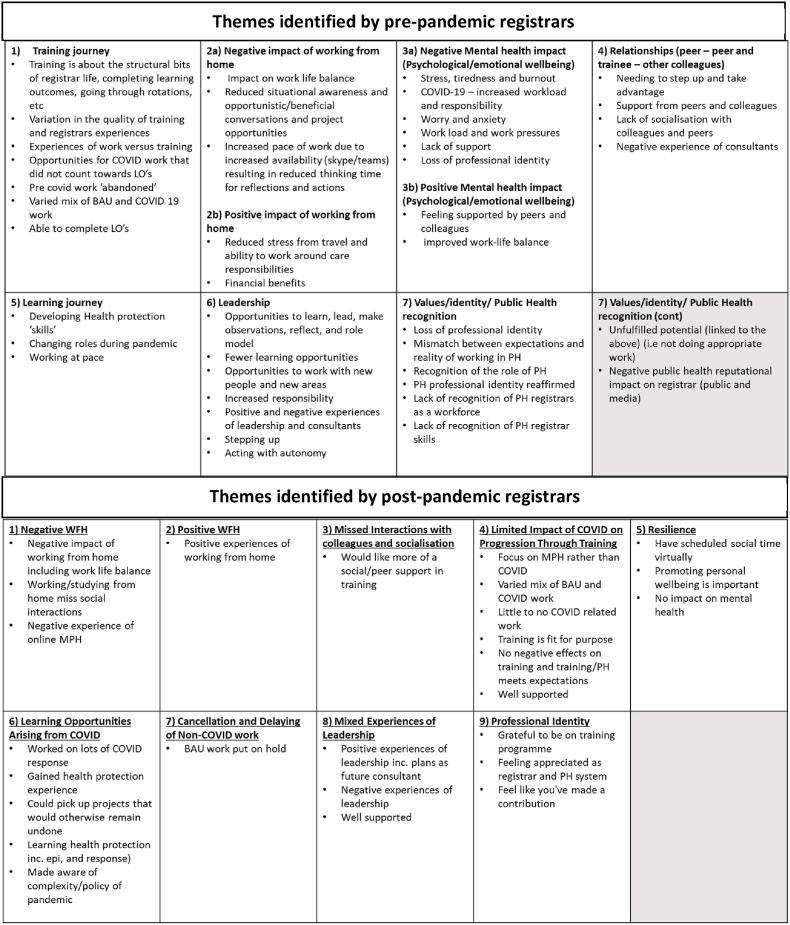
Themes identified in PAR 1.

Pre-pandemic Registrars identified eight themes (Fig. 2). The discussion generally highlighted negative experiences of training throughout the pandemic, with participants describing how their training was hindered by a large amount of health protection work and remote working. Negative mental health and wellbeing was a strong theme with Registrars reporting impacts on personal and professional lives.

Nearly two years after the start of the pandemic, difficulties remain in maintaining an effective work-life balance. Some positives were noted however, including examples of observed leadership. Most Registrars also found opportunities to fulfil leadership roles themselves, but whilst some enjoyed this task, others felt unprepared. Indeed, some felt that demonstrating and developing essential leadership skills was harder virtually.

There was an overall negative impact on relationships with fellow Registrars. An originally collegiate group had found it difficult to gather, affecting access to peer support. This was compounded by two new cohorts starting training remotely.

The final two themes related to recognition, with lack of acknowledgement ranking more highly than feeling appreciated. Generally, Registrars felt that they were underutilised during the pandemic with a smaller section feeling that their identity as public health professionals was reaffirmed.

#### 3.3.2 Post-pandemic

Post-pandemic Registrars identified 9 themes (Fig. 2). The two main themes were negative and positive impacts of remote working. Opinions on the effectiveness of virtual learning spaces were mixed with those who had spent much of their time studying for their Masters in Public Health (MPH) preferring to work from home compared to those that did not.

The theme of missed interactions highlighted that Registrars felt a lack of social interaction within the training programme. Registrars expressed that so far, the pandemic had a limited impact on their training. Most felt well supported and encouraged to be involved in non-COVID-19 response work. Some Registrars described pro-active efforts to limit the impacts of loneliness by scheduling social time virtually and promoting personal wellbeing. Registrars also experienced no significant impact on mental health because of remote working.

Registrars described increased health protection related learning opportunities arising from the pandemic; projects that would otherwise not have been available, and general learning about complexity of pandemic response. A theme felt by a minority was cancellation and delay of business as usual (BAU) work.

Experiences of leadership were mixed with some Registrars feeling they were able to observe good role models virtually whilst others felt they lacked leadership opportunities. The final theme was of professional identity and showed that Registrar's identities as public health professionals were reaffirmed during the pandemic.

### 3.4 PAR 2 findings (meta-themes)

#### 3.4.1 Pre-pandemic

**Fig. 3:**
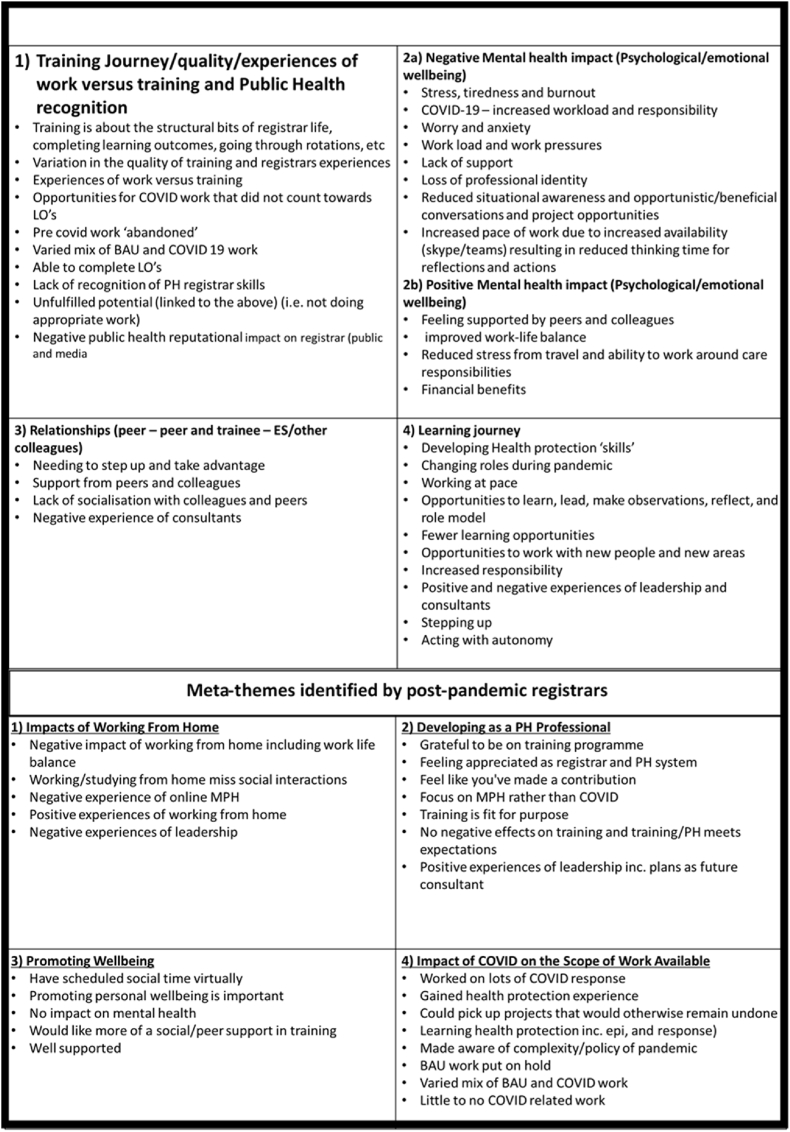
Meta-themes identified in PAR 2.

The pre-pandemic Registrar PAR group identified four meta-themes (Fig. 3). Cutting across these, was also the theme of positive and negative impacts of remote working.

##### 3.4.1.1 Theme 1: Lack of support

It was felt that the structural demands of Registrar life (including completing learning milestones), was not prioritised early in the pandemic and that doing so is an ongoing challenge for some. There was variation in the quality of training and educational supervision.

“Everything about training has changed (location, activities, access to support etc) yet the curriculum or expectations of us as Registrars has not. The land grab on your time is acute, trainers and colleagues do not always allow enough time for you to do all the admin stuff”

##### 3.4.1.2 Theme 2: Negative mental health impacts

There were emotional and psychological impacts experienced by Registrars due to increasing pressures and workload. This led to increased worry, anxiety, and risk of burnout.

“I have background anxiety that never goes away. I've never experienced this before”

A protective mental health factor was feeling supported by peers and colleagues.

“Training in a pandemic also isolates you from your peers and support group. I try and stay in touch on WhatsApp and attend training days where possible. Feeling part of a peer group is important for feeling connected and coping with the changes to training in a pandemic”

##### 3.4.1.3 Theme 3: The importance of personal and professional relationships and networks

Registrars identified the importance of feeling supported by peers and colleagues (a protective factor for mental health) and were concerned about the negative impact on the training experience of remote working.

“*The move to work from home has had a negative impact, both in terms of missing out on meeting new colleagues and seeing people in the office and feeling more isolated at home, and in feeling more isolated from other Registrars and not having that reassurance that I'm not alone in finding certain aspects of training challenging”*

##### 3.4.1.4 Theme 4: Learning dominated by COVID-19 and health protection

Registrars described roles predominantly in health protection and rapid changes to roles during the pandemic, leading to variations in learning opportunities. All Registrars had improved health protection proficiencies, potentially at the expense of other skills.

“*It feels it has moved more to the needs of the placement and that there is an underlying expectation some of your time will go to COVID-19 response regardless of if it is beneficial for your learning or not.”*

There were concerns about the impact on future progression for some due to lost learning opportunities.

“My projects were cancelled with pandemic response work taking over. I feel anxious and fearful of my future on the scheme and job opportunities on CCT'ing”

#### 3.4.2 Post-pandemic

The post-pandemic Registrars identified four meta-themes (Fig. 3).

##### 3.4.2.1 Theme 1: Impacts of remote working

Registrars experienced a mix of positive and negative impacts arising from remote working during the pandemic. These were distributed amongst the group, with a particular focus of its effects on work-life balance. Views that were expressed included an appreciation of the time gained in lieu of commuting and a feeling of being better able to manage time at home than in an office, counterbalanced by a sense of missing the ability to build relationships with colleagues.

“working from home has allowed me to slow down my pace of life, have more time for self-care and develop new hobbies. I like the freedom to work uninterrupted and get into the flow. I feel like I am more productive at home.”

“Working from home is isolating and you lose the normal social corridor conversations and networking opportunities that you might ordinarily experience under usual circumstances”

##### 3.4.2.2 Theme 2: Developing as a public health professional

Registrars expressed sentiments of finding work in public health to be fulfilling and rewarding. Some felt that they had been able to make meaningful contributions to their public health teams, through either pandemic response or BAU projects.

“There is a great public health community and I am glad to have joined the team”

The group felt that the training programme supported them well. It was recognised however, that with a focus during the first year of training on completing the MPH, post-pandemic Registrars had perhaps been less exposed to some of the service-related pressures experienced by the wider public health workforce.

##### 3.4.2.3 Theme 3: Promoting wellbeing

Registrars strongly felt they had been well supported during their time on the training programme by more senior public health professionals, and particularly by their Educational Supervisors. They recognised the importance of ensuring good personal wellbeing and felt that they had been able to make adaptations to ways of working to ensure this.

“I have also learnt to value the normal day to day social interactions with work colleagues”

Despite implementing initiatives such as virtual catchups, Registrars did still feel like they would benefit from face-to-face social interaction with colleagues.

##### 3.4.2.4 Theme 4: Impact of COVID-19 on the scope of work available

Most Registrars noted their work was primarily focussed on areas not directly associated with pandemic response.

“I have not at any time been pressured into contributing to large operational pieces of COVID response work, but instead been encouraged to work in other areas that will benefit my signing off of learning outcomes.”

Even when contributing towards work that was considered BAU, Registrars noted impacts arising from the pandemic, such as delays resulting from the de-prioritisation of non-COVID-19 work by organisations. Registrars appreciated having been able to observe and participate towards the wider systems’ response to the pandemic and felt they had not experienced difficulties in achieving required learning outcomes.

“I think also working in public health has given me greater understanding and insight of the pandemic situation as it evolves, and if I wasn't in public health perhaps, I might have felt more uncertain and as such anxious”

### 3.5 Similarities between the two groups

Some themes showed similarities such as a high ranking of the impact of remote working and reaffirmation of professional identity. Relationships between Registrars remained an important part of training with both groups feeling a lack of peer support. Both groups cited the new learning opportunities offered by the pandemic, with the pre-pandemic group being given opportunities to lead and the post-pandemic group learning about health protection and systems.

### 3.6 Differences between the two groups

There are multiple differences between the pre- and post-pandemic training groups, including the differing view of mental health impact, support, and remote working. Pre-pandemic Registrars described the pandemic as a difficult time in their lives, where mental, psychological, and emotional health had declined significantly due to the workload and need to work remotely. In contrast, most post-pandemic Registrars cited that there had been no impact on their mental health and a focus on wellbeing and looking after oneself was more prominent. For the pre-pandemic group, negative impacts of remote working ran throughout all themes. For the post-pandemic group, Registrars reported feeling well supported and held positive views of consultants and their training experience.

## 4 Discussion

### 4.1 Main finding of this study

The findings show a difference in training experiences during the pandemic for those who started training before the pandemic versus those who started training after. Some common threads ran throughout both groups, but it is of note that the general sentiment of the pre-pandemic group was a negative one whilst for post-pandemic it was positive.

### 4.2 What is already known on this topic

Views of the pre-pandemic group were captured in a previous publication [1], themes remained broadly similar showing no major change in the sentiment of the cohort regarding negative experiences of remote working. Learning continues to be impacted, with some Registrars thriving and others faltering. This sits in congruence with other research with public health Registrars, which found a mixed view amongst another Registrar cohort^1^ regarding impact of COVID-19 on learning outcomes. This research also supports our study findings, that remote working has negatively impacted mental wellbeing and opportunities to learn and shadow [11]. The GMC's 2021 National Training Survey highlighted an increase in burnout amongst trainees across all specialties and a swing towards negative answers to wellbeing questions post pandemic compared to before. Furthermore, 10% were concerned about progressing through training [12]. Research shows other specialties described negative mental health impacts due to the impact of COVID-19 on their training [13]. Some opinion-based literature has suggested that the main impact of COVID-19 is on procedure-based specialties [14], however our research would challenge this. Previous publications have suggested the pandemic experience as an opportunity to reconsider approaches to public health education [2,9]. Further comparison literature within this topic area was not found, which strengthens the need for this exploration.

### 4.3 What this study adds

This study follows the first original research into the impact of the pandemic on public health speciality Registrars training in England. In contrast to the previous paper, this study uses a novel, comparative methodology [5,6] to compare training experiences of Registrars who started training before the pandemic to those who started after. The addition of a question on mental health experiences highlighted negative impacts on mental health (including anxiety, loneliness, and burnout) for the pre-pandemic group and enables the mental health experience of this cohort to be situated within that of other specialties.

Pre-pandemic Registrars are concerned about poor achievement of learning outcomes due to continued pressure to work on pandemic response as well as a lack of varied opportunities. This coupled with a lack of appropriate leadership opportunities create the risk that Registrars will not be well prepared for consultant practice [2,4,10].

Post-pandemic Registrars generally felt well supported with no negative impacts on their mental health. For them, negative experiences of completing their MPH virtually meant a potential lack of knowledge of the foundations of public health. Lack of peer support in the early stages can result in lack of social connectivity as training progresses which could impact future mental health.

### 4.4 Limitations of this study

Participation in the research process was voluntary and therefore the views of all Registrars were not captured. Some Registrars who wished to participate were unable due to work pressures. Those in the post-pandemic group were in their first two years of training, which, given those at the start of training spend less time on service work and may experience less service level impacts, could have confounded the results.

Although the results are specific to this cohort of East Midlands Registrars, we anticipate that experiences would be similar for Registrars in other English regions (due to having the same curriculum, generally similar opportunities and previous research), and potentially the wider public health workforce. Whilst the sample was small, this still accounted for almost half of the Registrars in the East Midlands region and enabled us to gather rich insights, as is the aim of a qualitative approach such as PAR.

### 4.5 Implications for practice

The findings show the need for focussed support from supervisors and programmes to plan how outstanding learning outcomes will be met amongst those who started training pre-pandemic and feel learning outcomes were impacted. This could be through making targeted opportunities available to fulfil outstanding learning outcomes or extensions to training as appropriate, where COVID-19 has impacted ability to make sufficient progress. This may reduce Registrar anxiety. It is acknowledged this will not require implementation for all pre-pandemic Registrars as experience was varied and this support and planning may already be in place. Supported leadership opportunities should be provided to those who feel leadership was negatively impacted.

Much of the negative impact of COVID-19 centred around home working and mental health, due to isolation, reduced opportunity for spontaneous learning, issues with leadership experience and pressure. Implementation of wellbeing strategies (co-produced with Registrars), particularly those involving peer support opportunities would proactively address some of the issues regarding reduced networking for pre-pandemic Registrars and act as a protective factor for post-pandemic Registrars who over time may encounter similar negative impacts.

Novel ways to incorporate leadership skills whilst working virtually into the curriculum would be a key area for both groups, as well as finding innovative methods to increase spontaneous learning opportunities whilst working from home, addressing the removal of ‘corridor conversation’ type learning. Early leadership opportunities would increase resilience of training to disruption by acute public health emergencies. A balance of home and in person work should be offered based on the individual's needs.

In a post-pandemic world of home working, these findings are highly relevant as whilst the pandemic context may have become less acute, some environmental factors that impacted training still prevail, such as home working. If another pandemic were to occur, the findings here should be noted so that involved Registrars experience the varied learning experiences necessary to meet curriculum requirements as well as peer support opportunities to feel connected. Supervisors should have guidance for Registrars’ roles in acute emergency response and recovery to ensure findings are implemented.

Registrars are the future public health leaders and will be at the forefront of future public health emergencies. Their training should be prioritised to ensure a strong public health response in future.

## Funding

This research did not receive any specific grant from funding agencies in the public, commercial, or not-for-profit sectors.

## Ethical approval

Due to this work being undertaken independently of any particular institution, no formal ethical approval was sought. Participants reported on all consented to participation and inclusion of their data in the study.

## Declaration of competing interest

The authors declare the following financial interests/personal relationships which may be considered as potential competing interests: Some of the authors also took part in the survey. All authors were part of the East Midlands registrar cohort at the time research was undertaken.

## Footnotes

1 London, Kent, Surrey and Sussex.

Appendix A Supplementary data to this article can be found online at https://doi.org/10.1016/j.puhip.2022.100351.

## References

[1] Maile E.L., Horsley S.M., Dunn T. (2021 Sep 28). Initial impact of the COVID-19 pandemic on public health training: participatory action research to understand experiences in the East Midlands. J. Public Health.

[2] Ghaffar A., Rashid S.F., Wanyenze R.K., Hyder A.A. (2021 Apr 1). Public health education post-COVID-19: a proposal for critical revisions. BMJ Global Health.

[3] GMC (2021). https://www.gmc-uk.org/-/media/documents/somep-2020_pdf-84684244.pdf.

[4] Brisolara K.F., Smith D.G. (2020). Preparing students for a more public health–aware market in response to COVID-19. Prev. Chronic Dis..

[5] Xu A., Zare H., Dai X., Xiang Y., Gaskin D.J. (2019 Nov 1). https://journals.plos.org/plosone/article?id=10.1371/journal.pone.0225243.

[6] Lim H.M., Ng C.J., Teo C.H. (2021 Jun 1). Prioritising topics for developing e-learning resources in healthcare curricula: a comparison between students and educators using a modified Delphi survey. PLoS One.

[7] Braun V., Clarke V. (2006). Using thematic analysis in psychology. Qual. Res. Psychol..

[8] UK Government (2018). Data protection act 2018 [internet]. Legislation.gov.UK. https://www.legislation.gov.uk/ukpga/2018/12/contents/enacted.

[9] Resnick B.A., Mui P.C., Bowie J., Kanchanaraksa S., Golub E., Sharfstein J.M. (2021 Jan 1). The COVID-19 pandemic: an opportunity to transform higher education in public health. Publ. Health Rep..

[10] Czabanowska K., Kuhlmann E. (2021 May 1). Public health competences through the lens of the COVID-19 pandemic: what matters for health workforce preparedness for global health emergencies. Int. J. Health Plann. Manag..

[11] Hall L., Bisset K., Lynch L., Young Y., Ruggles R. (2022). Training during the COVID-19 pandemic: the experience of public health registrars in the London and Kent, Surrey, Sussex training programme. J. Publ. Health.

[12] General Medical Council (27 July 2021). https://www.gmc-uk.org/news/news-archive/doctors-burnout-worsens-as-gmc-report-reveals-pandemic-impact.

[13] Hussain R., Singh B., Shah N., Jain S. (2020 Dec). Impact of COVID-19 on ophthalmic specialist training in the United Kingdom-the trainees' perspective. Eye.

[14] Yu Christiaan, Mei Teh Bing (2020). Ar Kar Aung ‘COVID-19 significantly affects specialty training’. Intern. Med. J..

