# Supplementary material for: Comparing impacts of the COVID-19 pandemic on training of public health Specialty Registrars starting before or after its onset
Source: Public Health Pract (Oxf). 2022 Dec 17;5:100351. doi: 10.1016/j.puhip.2022.100351 (PMC9758068; doi:10.1016/j.puhip.2022.100351)
Supplement: Multimedia component 1 [file mmc1.docx]

Appendix 1: Delphi 1 survey questions

**Open questions**

1. How has your role changed (if at all) since the start of the COVID-19 response?
2. Please share in a few words what your role has been since the start of the COVID -19 pandemic whilst in training.
3. In terms of your training – what have been the opportunities presented by the COVID-19 response?
4. How do you feel about the role you have played in the pandemic to date?
5. How does your role in the pandemic match what you would have expected the role of a public health registrar to be?
6. How has training in public health during the COVID-19 pandemic affected your mental health/wellbeing (if at all) and what coping strategies have you used if applicable?
7. In terms of your training – what have been the challenges presented by the COVID-19 response?
8. In terms of your training – could anything have been/be done differently? How? What difference would this make?
9. What have you learned from the response?
10. How would this learning shape your role as a consultant/and or what would you do differently as a future leader?
11. Consent: I agree to my answers being used to inform a publication

**Demographic questions**

- Phase 1 (Pre part B) or Phase 2 (post Part B)
- Medic or non-medic
- Started training Pre COVID-19 or started training during COVID-19
- Ethnicity (“non-White”/”White”)
- Age (25-29, 30-34, 35-39, 40–44, 45–49, 50-54, 55-59, 60-64, 65-69)
