# Supplementary material for: Comparing impacts of the COVID-19 pandemic on training of public health Specialty Registrars starting before or after its onset
Source: Public Health Pract (Oxf). 2022 Dec 17;5:100351. doi: 10.1016/j.puhip.2022.100351 (PMC9758068; doi:10.1016/j.puhip.2022.100351)
Supplement: Multimedia component 2 [file mmc2.docx]

Appendix 2: List of protothemes

| Pre-pandemic registrars protothemes with count | |
| --- | --- |
| 67 | Negative impact of working from home including work life balance |
| 59 | Negative impact on mental health |
| 44 | Lack of training support |
| 43 | Training not prioritised due to COVID work/harder to tick off LO |
| 32 | Fewer learning opportunities |
| 31 | Positive experiences of leadership inc. plans as future consultant |
| 29 | COVID gives opportunity to work with new colleagues |
| 27 | Change in role during pandemic |
| 26 | More learning opportunities |
| 23 | Learnt a lot about health protection inc. communications |
| 23 | Cannot escape COVID19 |
| 21 | Shorter timescale projects/work at pace |
| 20 | Burnout (chronic workplace stress, lack of control) |
| 19 | Health protection positive experiences |
| 18 | Lack of recognition of PH |
| 15 | Negative experiences of leadership |
| 15 | Increased workload and responsibility as a result of COVID |
| 12 | Made a useful contribution to response |
| 12 | Tired or fatigued |
| 12 | Lack of socialisation with colleagues/peers |
| 11 | Worry/anxiety |
| 10 | Not made a useful contribution to response |
| 10 | Public health professional identity reaffirmed |
| 10 | Pre COVID projects abandoned |
| 9 | Frustrated at policy or processes |
| 8 | Difficult work and working practices |
| 8 | Feeling supported by ES, peers, etc. |
| 7 | Health protection negative experiences |
| 7 | Recognition of PH |
| 6 | Positive impact of working from home including work life balance |
| 4 | Positive experience of consultants |
| 4 | Loss of professional identity/miss match between expectations and reality of PH |
| 4 | Varied mix of BAU and COVID work |
| 3 | Promoting personal wellbeing is important |
| 3 | Positive impact on mental health |
| 3 | FPH curriculum needs review |
| 3 | Able to complete learning outcomes |
| 3 | Need to step up to take advantage and risk missing out/act with autonomy |
| 2 | Negative experience of consultants |
| 1 | No reassurance from Es, peers etc. |

| Post-pandemic registrars protothemes with count | |
| --- | --- |
| 33 | Positive experiences of working from home |
| 33 | Negative impact of working from home including work life balance |
| 27 | No negative effects on training and training/PH meets expectations |
| 21 | Would like more of a social/peer support in training |
| 19 | Little to no COVID related work |
| 19 | Learning health protection inc. epi, and response) |
| 18 | Positive experiences of leadership inc. plans as future consultant |
| 18 | Varied mix of BAU and COVID work |
| 11 | Focus on MPH rather than COVID |
| 9 | Bigger contribution to pandemic outside of training |
| 8 | Gained health protection experience |
| 7 | Negative experiences of leadership |
| 6 | Well supported |
| 6 | Working/studying from home miss social interactions |
| 5 | Recent transition from clinical to public health |
| 5 | Made aware of complexity/policy of pandemic |
| 4 | Could pick up projects that would otherwise remain undone |
| 3 | Negative experience of online MPH |
| 3 | Promoting personal wellbeing is important |
| 3 | Feeling appreciated as registrar and PH system |
| 3 | Feel like you've made a contribution |
| 3 | Training is fit for purpose |
| 2 | Grateful to be on training programme |
| 2 | Have scheduled social time virtually |
| 2 | No impact on mental health |
| 1 | BAU work put on hold |
| 1 | Worked on lots of COVID response |
